# In-person versus online delivery of a behavioral sleep intervention (Sleeping Sound^©^) for children with ADHD: protocol for a parallel-group, non-inferiority, randomized controlled trial

**DOI:** 10.1186/s12887-023-04329-y

**Published:** 2023-10-06

**Authors:** Maya K. Malkani, Andrew M. C. Sheridan, Alison J. Crichton, Romola S. Bucks, Carmela F. Pestell

**Affiliations:** 1 School of Psychological Science, University Western Australia, Perth, Australia; 2 Department of Paediatrics, Monash University, Melbourne, Australia; 3 School of Population and Global Health, University of Western Australia, Perth, Australia

**Keywords:** ADHD, Children, Intervention, Online, Randomized controlled trial

## Abstract

**Background:**

Children with Attention-Deficit/Hyperactivity Disorder (ADHD) often experience sleep difficulties such as difficulty initiating and maintaining sleep. Problem sleep may impact children’s daily functioning and behaviors and exacerbate ADHD symptoms. Most effective behavioral interventions to improve sleep are conducted in person, limiting accessibility to treatment for individuals in remote or rural communities or those who are unable to attend a clinic. This trial aims to assess the efficacy of delivering an established behavioral intervention online, Sleeping Sound with ADHD^©^, compared to a face-to-face delivery mode.

**Methods:**

This parallel group, non-inferiority, randomized controlled trial (RCT) will include at least 68 children, aged 5–12 years old with ADHD. Families of children will be recruited from private developmental and psychological clinics and social media, within the state of Western Australia (WA). Once written informed consent and baseline questionnaires are completed, families are randomized to receive the behavioral intervention either in-person or online via Telehealth services. The intervention targets the assessment and management of reported sleep problems, through two individual consultations and a follow-up phone call with a trained clinician. The sleep outcomes assessed consist of a parent-reported sleep questionnaire and actigraphy.

**Discussion:**

To the best of our knowledge, this is the first RCT to investigate sleep treatment modality for children with ADHD. If effective, clinicians can provide an evidence-based sleep intervention in an accessible manner.

**Trial registration:**

ANZCTR, ACTRN12621001681842. Registered 9 December 2021—Retrospectively registered.

## Background

Approximately 5% of children and adolescents are diagnosed with Attention-Deficit/Hyperactivity Disorder (ADHD), making it one of the most common neurodevelopmental disorders [1]. Symptoms, commonly inattention, impulsivity, and hyperactivity, must be present across multiple settings such as at home and school [2].

### Sleep difficulties in children with ADHD

Research examining the occurrence and association of sleep disturbances in children with ADHD suggests prevalence rates of 70% to 85% [3, 4]. Common sleep difficulties in children with ADHD include inconsistent and/or delayed sleep onset, bedtime resistance, sleep association, night-time waking, and excessive daytime sleepiness [3, 5, 6]. Sleep difficulties often negatively impact aspects of children’s day-to-day lives, and their families, more generally [7]. Sleep difficulties have been demonstrated to impair functionality across numerous domains such as ADHD symptom severity, life skills, and interpersonal relationships [3, 6]. In addition, child sleep disturbance has negative implications for their families, for example through caregiver stress and affect [3] and family quality of life [8].

The interaction of sleep disturbances and ADHD in children is complex and the directionality of the associated effects is unclear, i.e., ADHD can negatively impact sleep and disrupted sleep may exacerbate ADHD symptoms [9, 10]. Nevertheless, similarities between ADHD and disturbed sleep symptomology have been suggested, including reduced cognitive functioning and exacerbated undesirable behaviors such as increased bedtime resistance [11–13]. Disrupted sleep is common regardless of whether or not children use stimulant medication for their ADHD [13]. Previous research provides mixed findings regarding the impact of stimulant medication on sleep in children with ADHD. However, a meta-analysis of RCTs concluded that stimulant medication was associated with reduced sleep efficiency and sleep duration, as well as extended sleep latency [14].

### Treatment of sleep problems in children with ADHD

Treatment for sleep difficulties in children with ADHD can be two-fold; pharmaceutical and/or non-pharmaceutical. Increase of the naturally occurring hormone, melatonin, in the form of a supplement, has been shown to assist in the initiation and maintenance of sleep [15, 16]. Empirical evidence has identified the effectiveness of melatonin for children, especially those with neurodevelopmental disorders like ADHD (e.g., [15, 17]). However, children with ADHD often exhibit symptoms and behaviors that are not conducive to sleep initiation and/or maintenance. Therefore, behavioral strategies can be implemented either independently or in parallel with pharmaceutical treatment. Research using behavioral sleep interventions in children with ADHD has shown improvements in sleep, ADHD symptomology, and mood [18, 19]. Behavioral interventions for sleep often encompass components of healthy sleep practices, behavioral, and/or cognitive strategies. Intervention targets poor pre-sleep behaviors by providing structure, rules, and positive reinforcement at bedtime [20]. Some strategies may be employed more broadly across differing sleep problems, such as positive reinforcement and relaxation techniques [21], whereas other strategies may be implemented for specific sleep problems. For example, delayed sleep onset may be managed using bedtime fading, whereby the child’s bedtime is initially set at their later sleep onset time, then incrementally brought forward to a more appropriate time [22].

### Telehealth interventions

Telehealth, distanced, or online interventions may assist in reducing barriers to treatment access, such as residence in rural or remote communities, and associated travel time and costs [23, 24]. In recent years, there has been a significant increase in the demand for online or telehealth treatment given the rise in technology usage [25] and the worldwide pandemic, Coronavirus disease 2019 (COVID-19). Few studies to date have targeted treatment of ADHD in children via telehealth services, however, much of the research conducted is encompassed within The Children’s ADHD Telemental Health Treatment Study (CATTS, [26, 27]). CATTS is a 5-year RCT of evidence-based psychiatric and behavioral treatments for children with ADHD provided via telehealth. Behavioral treatment included therapist-delivered psychoeducation and caregiver behavioral training, telepsychiatry, psychoeducation, and/or caregiver parent training. Across studies, the project demonstrated effective treatment of ADHD via telehealth. Additionally, Xie [28] reported equivalent treatment effects of a group parent training program for parents of children with ADHD when delivered in-person compared to via videoconferencing. The study demonstrated significant parent-reported improvements in ADHD symptoms across both delivery groups and findings suggest the caregiver-accepted videoconferencing mode may be as effective as in-person training.

In a systematic review of telehealth interventions for sleep problems in children and adolescents (including, but not limited to, children with ADHD), evidence was found to support the use of telehealth interventions for childhood insomnia [23]. The review reported on ten studies, where intervention included general psychoeducation and/or behavioral skills training and noted that treatment for children often included caregivers as mediators. Behavioral change in children generally requires parental engagement, with parents acting as both the agents of change as well as providers of support via facilitating their child’s use of the intervention strategies. Intervention tools included written manuals, modules, and activities, as well as individually tailored feedback, delivered by web-based platforms [23].

Two studies have examined sleep treatment specifically for children with ADHD, whereby a behavioral sleep intervention was delivered in a distanced format, i.e., a manualized intervention with telephone consultations [29, 30]. The original case series study demonstrated positive findings [29] and allowed for further development of the intervention. Corkum [30] reported promising improvements in treated children’s sleep within groups of typically developing (TD) children and children with ADHD. The study surmised that 42.1% of the TD children and 41.7% of the ADHD children in the treatment group improved from the clinical to nonclinical range on the Children’s Sleep Habits Questionnaire (CSHQ) Total Sleep Disturbance Scale after intervention, compared to 15.8% and 0%, respectively, in the waitlist control group. This research indicates the potential for accessible, effective intervention. Currently, however, research into online behavioral interventions for children with ADHD and poor sleep is limited.

### Rationale and objective

We recently conducted a systematic review and meta-analysis assessing behavioral sleep interventions for children with ADHD [31]. Based on analyses conducted, the review suggested a brief, individualized intervention may be effective at improving sleep in children with ADHD. However, many of the studies reviewed conducted intervention in-person, meaning further evaluation of online treatment is required to increase the accessibility of effective intervention. Therefore, the objective of this project is to compare the treatment effects of an in-person versus online delivery of an established intervention to improve experienced sleep problems in children with ADHD.

## Methods/Design

### Study design

**Fig. 1:**
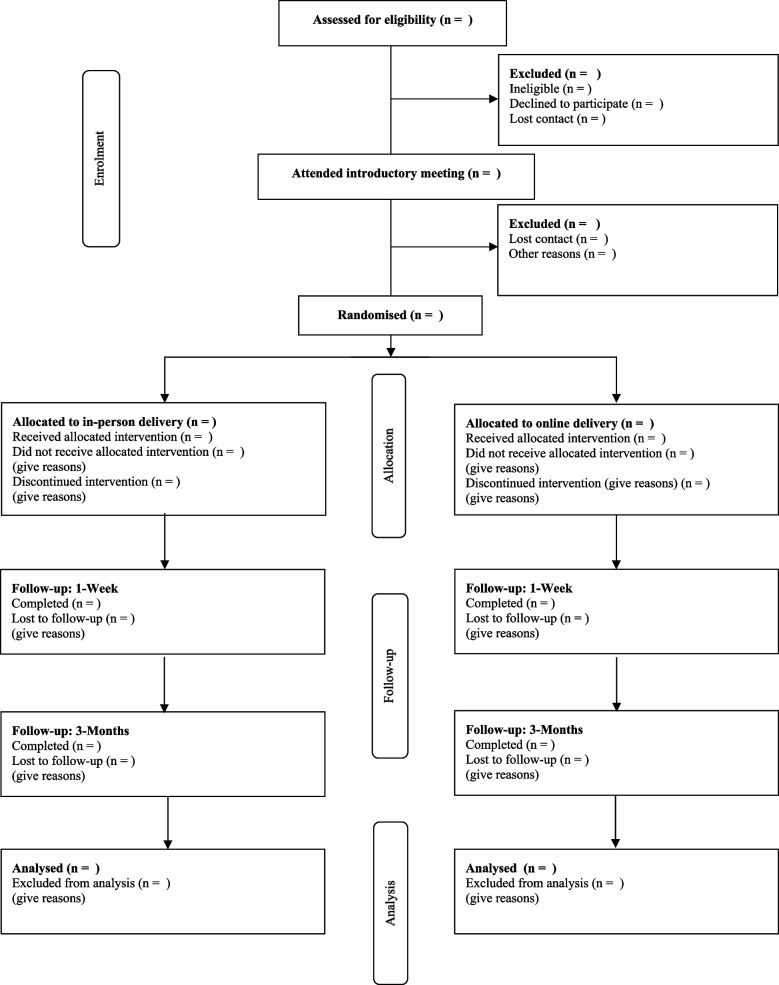
Participant flow diagram

This project is designed as a parallel-group, non-inferiority, RCT of a behavioral sleep intervention delivered online (telehealth) or in-person (see Fig. 1). The project will use Sleeping Sound with ADHD^©^ (referred to as Sleeping Sound for the remainder of this article, [32], an evidence-based behavioral sleep intervention for school-aged children with ADHD, shown to improve sleep when delivered face-to-face [18, 21, 24]. Recruitment and baseline data collection will occur between October 2021 and November 2022, and the intervention will be delivered from October 2021 to March 2023. The intervention will be conducted at the Robin Winkler Clinic (UWA).

The trial was registered with the Australian New Zealand Clinical Trials Registry (ANZCTR) on 9 December 2021; registration number ACTRN12621001681842. All procedures involving human participants were approved by the University of Western Australia Human Research Ethics Committee (UWA HREC), reference: 2021/ET000637. This study protocol adheres to the Standard Protocol Items: Recommendations for Interventional Trials (SPIRIT) checklist.

### Participants

Participant demographic parameters, such as child age, were sourced from the inclusion criteria of the Sleeping Sound project protocol [32]. Families of children aged 5–12 years, with a diagnosis of ADHD will be recruited within Western Australia. Due to the demographic characteristics of the sample, a greater number of male children is likely, however males and females will be targeted for recruitment equally.

By parent description, children will experience at least one of the following sleep difficulties, singularly or in combination: delayed sleep phase (shift in sleep–wake cycle resulting in a later bedtime and wake time);insomnia and/or night-time waking (marked difficulty initiating and/or maintaining sleep); sleep onset association (e.g., falling asleep is associated with an object or person); limit setting (resistance or refusal at bedtime); night-time anxiety (specific night time fears and/or worrying when attempting to sleep).

### Inclusion and exclusion criteria

Eligible families will include children with both a pre-existing diagnosis of ADHD and moderate/severe sleep difficulties by parent/guardian report as per the sleep problems listed above. For children with suspected ADHD awaiting paediatric or psychiatric review, follow-up contact will be made to discuss eligibility post-appointment. Children will be excluded from the study if they: (a) do not speak English; (b) have a diagnosed intellectual (IQ < 70) or other severe cognitive disability; (c) have a current diagnosis of obstructive sleep apnoea or narcolepsy; or (d) are currently receiving sleep treatment elsewhere, for example at a specialist sleep clinic. Children taking ADHD medication with sleep inducing qualities or taking melatonin will not be excluded. However, it will be recommended that use and dosage remain consistent throughout participation, with this information and any reported changes recorded on the sleep diary provided during data collection.

### Recruitment and enrolment

Private clinics in Perth, WA will be contacted for recruitment assistance in a manner deemed appropriate by the clinic, for example through placement of flyers within the clinic and/or social media circulation of recruitment material, such as newsletters. Relevant clinics include those offering psychology, paediatric, and/or multidisciplinary services. Further, social media advertisements will be targeted at families of children with ADHD. The recruitment flyer will direct families to contact one of two researchers associated with the project to register their interest. Families will then be contacted to arrange a brief screening call to assess eligibility.

If deemed eligible after screening, families will be provided with participant information sheets (parent / legal guardian and child) and invited to attend an introductory meeting with investigator MM, either in-person or online. The introductory meeting, attended by the child’s parent / legal guardian, involves enrolment processes such as acquiring informed consent and baseline questionnaire completion. The child themselves are encouraged to attend, however, this is not a requirement to reduce scheduling conflicts and impact on school attendance. A log of introductory meeting dates and child attendance will be kept.

### Consent to participate

Written informed consent from a parent or legal guardian will be obtained by investigator MM. Information sheets and signed consent forms will be provided for all participants involved in the trial. Children’s written informed assent will be sought from either MM, if the child is present when obtaining parent / legal guardian written consent, or by the child’s parent / legal guardian. The witness of the child’s written informed assent will also sign the assent form. Additionally, children’s verbal assent will be obtained by the treating clinician at the first intervention consultation session. Consent will also be sought from children and their parent / legal guardian to collect data for ancillary studies. Additional questionnaires completed will be used to explore children’s comorbid disorders and symptomology (Conners Comprehensive Behavior Rating Scale, [33]), general family functioning (McMaster Family Assessment Device, General Functioning subscale, [34]), and children’s parental attachment by parent-report (Attachment Insecurity Screening Inventory, [35, 36]) and self-report (Security Scale, [37]).

### Sample size

A power analysis using 3-month follow-up sleep outcome statistics from Hiscock [18], comparing intervention versus treatment as usual, was conducted in G*Power 3.1 [38]. To detect a large treatment effect (0.8) at 3-month follow-up, with 80% power and a two-sided significance level of 0.05, we require 26 children in each arm. Hiscock [18] reported a 28.3% participant loss at their 3-month follow-up. Therefore, we will allow for 30% attrition over the follow-up period, requiring 34 children in each arm, to a total of 68 participants.

### Randomization

Upon receipt of written informed consent and baseline surveys, investigator MM will randomize participants to either the in-person or online intervention delivery group. To achieve balanced characteristic distribution across the trial arms, covariate adaptive randomization will be implemented. Three covariates will be used in blocks of four; sex at birth, age, and presence of diagnosed comorbid psychiatric disorders. The randomization matrix will be generated by investigator MM prior to commencement of participant enrolment. A non-populated copy of this matrix will be kept, to ensure integrity and fidelity. All families will be contacted via email to inform them of their delivery allocation. Independently conducted randomization, allocation concealment, and outcome assessor blinding is not achievable due to limited resources. Given the nature of the study design, neither participants nor clinicians delivering the intervention can be blinded to delivery allocation.

### Intervention

The details of Sleeping Sound were published previously [32]. The intervention schedule consists of two, 50-min sleep consultation sessions for the child and parent^1^ and a follow-up call with the parent, each held two weeks apart. Duration of the follow-up phone call may vary; however, a range of 3–30 min was reported in Hiscock [18]. The consultations will occur with a trained clinician (provisional psychologists at UWA; n = 16). All clinicians will deliver the intervention across both delivery modes. The clinicians will be trained within one three-hour session, with adherence to the Sleeping Sound training manual. The training session will include information regarding normal sleep, sleep cues and cycles, healthy sleep practices, and behavior management strategies aimed at improving sleep. During the training session, case scenarios will be presented to the clinicians to support their understanding of diagnostic formulation and treatment plan development. Once the intervention phase has begun, clinicians will participate in weekly supervision sessions with investigator (AS), an experienced clinical psychologist and clinical neuropsychologist, to monitor adherence to the Sleeping Sound intervention by reviewing assessment content, discussing clinicians’ case formulations and treatment recommendations, and (for a subset of sessions) viewing video recordings. Record keeping, aided by a standardized consultation form, will include the presenting sleep problem(s), characteristics of the child, such as medication use and comorbid conditions, and current bedtime routines. The standardized consultation form will also include information regarding intervention adherence, such as deviations from the schedule due to illness. Treatment fidelity will be assessed via: (a) the number of participants who attended at least one consultation session; (b) the number of consultation forms completed; (c) the number of participants that attended each consultation session; (d) duration of consultation sessions; (e) average number of diagnoses per child; (f) average number of intervention strategies provided for each child.

The first consultation session will provide the families with psychoeducation about appropriate sleep, sleep cycles, and healthy sleep practices. A treatment plan tailored to the child will be developed based on the family’s identified goals for treatment and the clinician’s formulation of the child’s sleep problems. Families will be offered a range of appropriate treatment strategies and can preference strategies. Parents will also be asked to complete a sleep diary between the two consultation sessions. The second session includes progress monitoring, review of the sleep diary, strategy reinforcement, and troubleshooting. The follow-up phone call will provide the family with the opportunity to discuss progress, troubleshoot and reinforce strategies, or ask residual questions after the two consultation sessions. Any clinical recommendations not within the scope of the trial will be provided to the families after the final post-intervention data collection.

### Data collection

Subjective and objective data will be collected using a parent-reported sleep questionnaire and actigraphy respectively. All outcomes are measured at baseline and two post intervention time points (1-week and 3-months post intervention). The study will collect demographic information from the child’s parent or legal guardian. Medical information, such as known medical diagnoses, and a qualitative summary of the child’s experienced sleep problem(s) will also be collected.

Participants will be free to withdraw from the study at any time, for any reason and without consequence. Once a child is randomized, every reasonable effort to follow the child for the entire study period will be made. Non-adherence to the schedule of follow-up assessment will be recorded. Potential reasons for delay include slow response from families, illness, and COVID-19 related impacts. To allow for potential delays, data collection will be permitted within the ranges of 1–3 weeks post intervention and 12–16 weeks post intervention.

#### Primary outcome

The Children’s Sleep Habits Questionnaire (CSHQ, [39]) is a validated 33-item parent-reported measure of children’s sleep difficulties (α = 0.79). The measure assesses problems of initiation and maintenance of sleep, as well estimates of bed and wake times and total sleep time both with and without naps.

#### Secondary outcomes

The ActiGraph wGT3X-BT (ActiGraph Corporation) is a non-invasive monitoring device worn around the non-dominant wrist for seven nights at each data collection period. Measured body movements during sleep will be used to determine sleep and wake periods, providing the analysis variables of sleep duration, sleep onset latency, and sleep efficiency. Along with an instruction sheet, a sleep diary will be provided for parents to record information such as time in bed, sleep initiation time, and morning awakening time, etc. Parents will be prompted to include information such as time melatonin is taken (if any), abnormal night or behavioral activity, and illness.

### Statistical methods

The in-person treatment delivery arm will be compared against the online treatment delivery arm for all analyses. Sleep outcome data at baseline will be compared against the one-week post-intervention and the three-month post-intervention scores.

An ‘intention-to-treat’ analysis will be conducted on the primary outcome data. As a comparator, we will also report a per protocol analysis. Missing data will be assessed using Little’s MCAR test, with the implementation of an imputation model, such as multiple imputation or expectation maximization, if required. If imputation of missing data is required, the imputation method will be selected based on the percentage and pattern of missingness. After data preparation is complete and an analysis dataset has been created by investigator MM, an independent researcher will de-identify the data by removing information such as participant age, sex, medication use, and presence of comorbid disorders. The independent researcher will also create a dummy variable for delivery group (e.g., Group 1 and Group 2), codes for which will be concealed from MM. This will result in a locked dataset for analysis of intervention effects whereby MM is unable to discern the delivery mode of each group. Analyzed data will include mean scores of the aforementioned actigraphy variables and the total sleep score provided by the CSHQ. Linear mixed modelling will be used for both primary and secondary analyses, comparing treatment effects between intervention delivery modes and intervention effectiveness respectively.

### Data management

Data management of the trial will comply with the WA State Records Act and Good Clinical Practice (GCP). All trial questionnaire data will be entered electronically, via Qualtrics XM, by a parent / legal guardian of the participant and automatically coded. Consistency and range checks will regularly be completed, and data will be transferred to the project dataset. Actigraphy data will be removed from the device and stored after each data collection period. Data codes will be concealed by MM from the remaining members of the investigatory team until after the creation of a locked analysis dataset.

### Monitoring

This project is sponsored by UWA, under the care of the site clinical trials and human ethics officer. Occurrence of any adverse events will be documented and reported to the trial principal investigator and the trial sponsor.

### Confidentiality

Potential and enrolled participants’ personal information is to be collected by one investigator and will be kept strictly confidential. All data will be deidentified and enrolled participants will be allocated an identification code. Physical documents will be stored securely at the study site, in a locked file cabinet in an area with limited access, to be digitized and destroyed. All electronic databases and documentation will be secured within password-protected systems. The participant identification code list that links codes to other identifying information will be stored separately within password-protected systems. The requirement of keeping strict confidentiality was discussed verbally with all site staff.

### Dissemination policy

A plain language summary of the final trial results will be provided for trial participants at the completion of the project. Further, the results will be submitted for publication in peer-reviewed journals.

## Discussion

Sleep problems in children with ADHD are commonly reported [6, 11, 13]. Sleep difficulties can lead to increased daytime sleepiness, exacerbated ADHD symptoms, and impaired personal and family functioning [3, 6, 9]. In addressing these problems, current research has frequently demonstrated the effectiveness of in-person delivery of behavioral sleep interventions. Additionally, a small body of research has supported the use of online or 'distanced' behavioral interventions for children with ADHD. Telehealth services support treatment access for children and families living in remote or rural areas, caregivers balancing work commitments and/or siblings, and those with medical vulnerability. We believe that this project is the first RCT to investigate the efficacy of a behavioral sleep intervention delivered both in-person and online (telehealth) for children aged 5–12 years with ADHD. With the increasing utility and accessibility of telehealth services, it is important to augment current research by examining the efficacy of evidence-based interventions when delivered in an online format compared to in-person delivery. If efficacious, the resulting trial will support the use of Sleeping Sound delivered via telehealth, allowing for increased treatment accessibility for children with ADHD and their families.

## Data Availability

Not applicable.

## Abbreviations

ADHD: Attention-Deficit/Hyperactivity Disorder
CATTS: The Children’s Telemedicine Treatment Study
COVID-19: Coronavirus disease 2019
CSHQ: Children’s Sleep Habits Questionnaire
GCP: Good Clinical Practice
HREC: Human Research Ethics Committee
RCT: Randomized controlled trial
UWA: University of Western Australia
WA: Western Australia

## Footnotes

1 Or legal guardian.
